# Changing perspectives in the training of endoscopic ultrasonography in Asia

**DOI:** 10.1002/jgh3.12587

**Published:** 2021-06-04

**Authors:** Chieh‐Sian Koo, Constantinos P Anastassiades, Khek‐Yu Ho

**Affiliations:** ^1^ Division of Gastroenterology and Hepatology National University Hospital Singapore

**Keywords:** Asia, education, endosonography

## Abstract

Training of endoscopic ultrasound (EUS) in Asia faces challenges of the ever‐increasing demand for skills to handle a growing range of both diagnostic and interventional EUS procedures, and a continual shortage of EUS training programs. To keep up with the pace of development in EUS, more short‐term EUS programs have been conducted across Asia in recent years. In this aspect, the Asian EUS Group (AEG) has taken the lead to fast‐track the dissemination of EUS knowledge and skills across Asia through its multinational network of training centers. AEG's programs are brought to wherever there is demand. Its versatile modular structure allows the program to be easily customized and scaled up or down to align to local needs, making it highly adaptable to the changing and varying needs in different countries. Even with the current pandemic situation, it has been able to continue its training efforts through the use of technology, including webinars, and live case demonstration.

## Abstract 2

Endoscopic ultrasound is a key technique to have in the endoscopist's armamentarium for both diagnostic and therapeutic purposes, but the training is challenging and opportunities are limited, especially in Asia. We describe the shifts that have been taking place in the Asian landscape in order to accommodate the needs of the trainees.

## Introduction

Endoscopic ultrasonography (EUS) was initially introduced as a diagnostic tool for the diagnosis and staging of various gastrointestinal malignancies, but it has rapidly evolved in the past decade. Continued innovations have pushed the frontiers of what endosonographers are capable of performing. This began with the advent of fine needle aspiration, but has quickly progressed to more complex, therapeutic procedures such as celiac plexus neurolysis, pancreatic pseudocyst drainage, bile duct and gallbladder drainage, treatment of gastric varices, palliative gastro‐enteric bypass, pancreatic tumor ablation, and is now even expanding into the realm of endohepatology.^1^ The unique ability to perform both diagnostic and therapeutic interventions has made EUS a highly sought‐after tool for endoscopists to have in their armamentarium.

## Training in endoscopic ultrasonography

It comes as no surprise that interventional EUS and training competency for advanced endoscopy have been recently identified as two of the top 10 advances in gastrointestinal endoscopy.^2^ Despite increasing worldwide enthusiasm for EUS and its expanding applications, the path to attaining proficiency is long and arduous. Operators must become familiar with unique maneuvers using the various types of echoendoscopes and their accessories; be aware of the indications and risks involved; and be able to recognize and differentiate normal and diseased anatomical patterns based on ultrasonographic images. It is considered one of the most technically challenging procedures for the endoscopist to learn, as it requires the development and mastery of technical, cognitive, and interpretative skills. For the trainee, EUS is different to other procedures and more difficult to learn alone. This is because the ultrasound images are foreign to most general endoscopists. Furthermore, many aspects of the EUS procedure are often counterintuitive and require the opposite of what would normally be done with regular endoscopy, that is, remove all the air, make close contact and press up against the gastrointestinal wall, and use ultrasound landmarks instead of endoscopic landmarks. Finer and slower movements are required, and the acquisition of necessary hand–eye co‐ordination to trace structures can be challenging because of deviation from the traditional up/down orientation.^3^

The quality of EUS is directly proportional to the training, skill, and experience of the practitioner. The steady emergence of new interventional EUS techniques adds yet another layer of complexity in the training of aspiring as well as more established and experienced endosonographers. As soon as endosonographers acquaint themselves with contemporary EUS practice, ongoing and rapid developments in new technology, equipment, and accessories leave them in ever‐lasting pursuit of new knowledge and skill acquisition. Over the last few years, even experienced endosonographers have found themselves in need of keeping up with a variety of transitions, such as moving from mastery of radial to linear echoendoscope use; and from plastic stents and cystotomes to lumen‐apposing metal stents.

Specialized training via a formal advanced endoscopy fellowship has always been believed to be the best way for trainees to learn the art of EUS. This has traditionally been based on an apprenticeship model, where learning is primarily from direct patient encounters under the guidance of an expert teacher. In order to achieve this, the training center should have sufficient case volume and breadth to provide adequate training. For example, in its latest privileging and credentialing guidelines, the American Society for Gastrointestinal Endoscopy (ASGE) recommends an increase to 225 EUS procedures as the minimum number to be performed before assessment of competency and granting of credentialing or privileges.^4^ Didactic education should also be provided regularly, and this can be done via books, videos, lectures, and other media. Ideally, training should be carried out in a multidisciplinary environment together with surgeons, oncologists, pathologists, and radiologists, so that budding endosonographers understand their role in patient management.^5^ The typical fellowship runs over a period of 12 months, and the eventual aim is for the trainee to have acquired enough knowledge and skills to be able to perform procedures independently and safely.

## Training challenges in Asia

Formal fellowships remain the only form of accreditation for EUS education in Asia. Similar to other regions, the ideal training program is a well‐structured and balanced one that combines supervised hands‐on tutelage, scholastic learning, and self‐education at centers of excellence. Unfortunately, the lack of dedicated high‐volume centers and a shortfall of qualified mentors have resulted in a serious dearth of training opportunities in Asia. In the past, structured training programs in Asia were virtually nonexistent except for in Japan, Korea, and Taiwan. Endoscopists who were keen on training in EUS had to resort to either independent learning or relying on training programs at overseas centers of excellence, from which only a privileged few could benefit. Indeed, the lack of formal EUS training programs and opportunities in the Asian‐Pacific region was highlighted as an area of concern in a 2006 survey of endosonographers in the Asian‐Pacific region (excluding Japan). This showed that nearly half of all respondents were self‐taught, despite most agreeing that a formal fellowship with a minimum number of supervised procedures was necessary before competency in EUS could be achieved.^6^ The need to overcome learning curve issues enhance the acquisition of more advanced EUS knowledge and skills, and reduce the number of procedures required to gain proficiency was further illustrated in another Asian EUS study, which demonstrated wide variations in the interventional EUS practice of pancreatic pseudocyst drainage.^7^

The situation has improved in recent years, with a substantial increase in the number of EUS fellowships being offered in Asia. Creative alternatives in the form of observership attachments and short courses have also helped with training. However, significant training challenges remain and programs have to increasingly cater to both complete EUS novices as well as more experienced endosonographers looking to upgrade their skills, without compromising on the quality of practitioners that are produced. There are still insufficient slots to cater for all endoscopists who may be interested in further EUS training.

## Adapting to the challenges

The Asian EUS group (AEG) was established to meet the ever‐increasing demand for EUS training amid the continued shortage of training programs. First formed in 2012, this nonprofit professional interest group consists of regional experts who have taken the initiative to promote and accelerate the delivery of EUS knowledge and skills across Asia. However, Asia is a heterogeneous area with a diverse range of cultures and resources, and a uniform training model may not be suited for widespread implementation in the region. As such, new adaptive training models have been designed that are readily configurable to fit into the local context wherever necessary.

Structured short‐term education programs have been set up across Asia through AEG's multinational network of training centers. These programs are intensive, of a shorter duration, and without formal preceptorship. They consist of multiple modules and courses that typically include lectures, skills demonstrations by expert endosonographers, and hands‐on learning using simulators. These have been tailored to provide training across basic to advanced levels in order to meet specific learning outcomes. By matching the strengths of each training center to the trainees' needs, AEG has been able to scale up training in Asia and help propagate the dissemination of EUS skills in the region.^8^ More than 1400 endosonographers have been trained across such teaching programs, which have been successfully conducted across Asia, including China, Hong Kong, Japan, Korea, Taiwan, Vietnam, Thailand, Myanmar, Philippines, Malaysia, Singapore, Indonesia, India, Pakistan, Sri Lanka, and Saudi Arabia. The knowledge, skills, and competency that trainees have with regards to EUS have been shown to improve significantly after undergoing such programs. Such structured training courses appear to be an effective way of imparting EUS knowledge and skills to aspiring endosonographers in the Asian region.^9^ While these courses are neither as rigorous as full‐time fellowship programs nor designed to replace such programs, they do offer the advantage of flexible learning and help serve as a complement to the currently available training. While the AEG identified this regional void in Asia two decades ago, the urgent need to somehow satisfy community practice demands and train practicing endoscopists is now also being recognized in regions where EUS has traditionally been well established. On the other side of the Pacific, ASGE has been recently planning to launch a combined online and hands‐on training program in diagnostic EUS in the United States. This is targeted at an audience of practicing endoscopists who graduated from their basic endoscopy training 5 years ago or longer.^10^ Here, we elaborate further on the various means through which AEG has championed and adapted new approaches into their teaching programs across Asia, some aspects of which may have applicability in the rest of the world as well.

### Didactic learning and shift to virtual reality

The practice of EUS requires a deep understanding of the normal anatomy and the ability to visualize and interpret pathological abnormalities from ultrasonographic images. Gaining theoretical knowledge is equally important to being able to conduct the practical endoscopic examination. Theoretical knowledge acquisition is usually achieved through a didactic approach using learning aids such as textbooks, lectures, atlases, journal articles, and videos.

There has been an increasing shift in recent times to technology‐based forms of learning, which have the advantage of rapidly disseminating knowledge across a much larger group. New and better learning strategies need to be developed to cater to the newer generation of trainees, especially the millennial trainees, who perceive interactive technology as an important learning trait.^11^ This shift is even more important to embrace especially now in the context of the ongoing COVID‐19 pandemic. Traditional educational pedagogies are becoming outdated and unfeasible given the ongoing social restrictions that have to be imposed, and alternative models have to be adopted. The “flipped classroom” model is one such example, where trainees are provided with preparatory educational material that they can peruse at their own leisure prior to the teaching session. The aim of the teaching session then shifts to discussion, synthesis, and application of the provided material. This has been shown to improve knowledge acquisition, and has been used in pediatric gastroenterology teaching with positive outcomes.^12^

Similarly, AEG has adapted by moving the field of didactic learning to a virtual environment. Regular webinars and virtual conferences have been organized that bring expert speakers from around the region together to provide state‐of‐the‐art talks on their respective fields. This is directed to the level of the target audience, but generally encompasses the fundamentals, principles, and various clinical applications of EUS. Live endoscopy sessions and skills demonstrations by experts are now also increasingly carried out virtually through live broadcasts. This format allows trainees to have easy access to a wide range of faculty members, providing a platform for discussion and enhancing learning.^13^ In parallel, social media has also risen as an invaluable tool for augmenting individual learning. The AEG Facebook page has been used to quickly spread information about upcoming events and post educational infographics or video clips of endoscopic procedures with commentary. These are unique methods for engaging a large number of participants in interactive discussions, and are valued for their flexibility, speed, and reach.^14^

In addition to disseminating theoretical knowledge through these platforms, AEG has been a leader in creating novel EUS knowledge and training paradigms with the potential for integration into both EUS practice and training. For instance, AEG collaborators have demonstrated the feasibility of a complete pancreatobiliary linear endoscopic ultrasound examination from the stomach alone as opposed to the conventional examination scheme, which incorporates duodenal stations.^15^ This EUS examination format was shown to require a significantly shorter procedure time. This paradigm may be attractive for EUS training purposes because the procedure can be performed with the versatile linear rather than radial EUS instrument in case fine needle aspiration or biopsy needs to be performed, while the safety of the procedure may be increased due to avoiding access to duodenum, which may be associated with higher perforation risk. Such novel concepts are useful and, following possible validation studies, may be able to be applied in regular EUS training programs as well as shorter, structured courses organized by AEG and others.

### Simulators

Simulators occupy a key step between didactic learning and hands‐on experience with real patients. They allow trainees to safely build a basic framework of skills, reinforce skill maintenance, and shorten the learning curve for training of new techniques.^16^ A variety of simulators exist. Mechanical models such as phantoms or ex‐vivo animal models use a combination of materials and/or explanted animal organs to mimic human organs and generate realistic EUS images and conditions. These are easy to use and transport, readily configurable, and are relatively cheap. While the haptic feedback and characteristics of the imitation tissue are suboptimal, this method still remains useful for learning the technical aspects of a procedure. Live animal models are the most realistic simulators and are a good option for trainees looking to enhance their EUS skills.^17^ There is excellent anatomical resemblance to human anatomy, and the tactile feedback is nearly identical to that of human tissue. However, these models are expensive, difficult to set up, and can be associated with ethical controversies as well as cultural controversies applicable to the Asia‐Pacific region (such as the use of pig models, which could be unsuitable for many Muslim attendees). Computer‐based simulation models such as the EUS‐Mentor, Simbionix, and Voxel‐Man have been developed, which can provide detailed anatomic animation of EUS images to aid with image interpretation and faster learning. Unfortunately, availability and cost are major issues with these models. Even though available training resources in Asia can be limited, AEG has managed to continue to arrange physical workshops involving supervised hands‐on tutelage using animal models and phantoms under the necessary precautions even during the COVID‐19 pandemic.^18^ Furthermore, cultural obstacles have been successfully overcome by staying attuned to regional sensitivities, for example, replacing swine models with goat models for Muslim attendees.^17^

The creation of innovative models for practical teaching is of particular importance when it comes to interventional EUS. These procedures tend to be of a higher risk and associated with potentially devastating complications. It is essential for endoscopists to have performed sufficient cases to ensure competence, but this can be difficult to achieve unless the trainee is fortunate enough to be enrolled in a dedicated high‐volume training center. Even then, opportunities for teaching and training are limited because of low‐case volume for such procedures at most centers. The real need to improve structured training and successfully impart knowledge on beyond‐the‐basics, high‐interest yet low‐volume and high‐risk specific steps and maneuvers has similarly been highlighted in other areas of training in therapeutic endoscopy.^19^ AEG recently funded the creation of an advanced model for training of EUS‐guided biliary drainage to help bridge this gap. The novel Mumbai EUS 3D model, a bile duct prototype created by stereolithography 3D printing, gives trainees the opportunity to safely learn all of the steps involved in bile duct drainage using relatively realistic EUS and radiographic images.^20^ Subsequent evaluation of this novel, hybrid model showed that it successfully replicates situations encountered during EUS‐guided biliary drainage procedures. Within 10 days of training completion on the model, a significant number of trainees reported successful performance of this complex procedure independently.^21^ Ultimately, the role of simulators still needs to be clarified by further studies into the efficacy of EUS training models on technical and clinical outcomes. Simulators are, therefore, an adjunct to in vivo training and do not obviate the need for in vivo training or proctored cases.^22^ Such models are nevertheless useful to help EUS trainees gain understanding and skill in EUS procedures before embarking on performing the procedures in real patients.^18^

## The future of EUS training in Asia

A fundamental shift is occurring at all levels of medical training across the globe. Competency in endoscopy has historically been assessed based on the trainee completing an arbitrary number of procedures. However, reliance only on minimum procedure volumes is unreliable as trainees can have vastly different learning curves, and the number of procedures needed to achieve competence can fluctuate widely between different individuals.^23^ A move towards competency‐based education should be adopted to ensure that trainees have attained the necessary technical, cognitive, and integrative skills that are required for safe and independent performance of EUS. How then, can competency be properly assessed? Accurate and validated objective skill assessment tools are needed to monitor the learning curve of trainees. For example, the EUS and ERCP Skills Assessment Tool (TEESAT) has been endorsed by ASGE as a tool with strong validity evidence that facilitates assessment and grading of technical and cognitive EUS skills. It can be used in a continuous fashion throughout training to assess competence.^24^

Nevertheless, there is still great value in understanding the minimum number of EUS procedures required for an average trainee to achieve competency, and the idea should not be completely abandoned. As most existing EUS training programs do not have well‐defined curricula, being able to establish standardized syllabi and minimum standards will help to ensure adequate training and to facilitate assessment of trainee competence.^25^ A 2019 study using cumulative sum analysis of learning curves showed that the average EUS trainee required approximately 225 cases to achieve competence.^26^ Training programs can use this and other similar studies to establish the minimum standards required for case volume exposure, to guarantee that graduating trainees will able to meet all the necessary quality indicators for EUS independently and consistently.

This transition from a volume‐focused model to a competency‐based model is of particular importance in Asia. The lack of formal training opportunities has resulted in AEG's modular short‐term training programs being widely embraced, as they provide an alternative pathway to gain EUS knowledge and skills.^27^ While many have benefited from the open‐ended model of learning that is offered in AEG programs, it can still be challenging for trainees to achieve the required numbers needed to be internationally recognized and credentialed. The training programs may be able to adopt the tools used to assess competence to streamline educational content, individualize trainee learning plans, and ideally enhance learning outcomes. Having a standardized method of assessing competency would also allow graduates from these alternative learning pathways to be recognized and subsequently undergo apprenticeships with senior EUS practitioners for more hands‐on experience and tutelage.

In view of the significant developments in endoscopic training and competency taking place, leaders in endoscopic programs across the United States convened in 2017 to discuss the latest endoscopic training techniques and methodologies.^28^ It was acknowledged that endoscopic trainers fall into different teaching style categories and it was recommended that formal training of endoscopic teachers be undertaken to reach a level of “conscious competence” in order to maximize the benefit to trainees.^2^ Along these lines, the development and availability of “train the trainer” programs are gaining momentum. AEG has stayed well ahead of the curve in regard to EUS trainer concepts and has already been taking steps to ensure the longevity of EUS in Asia by embarking on a series of such train‐the‐trainer programs. This is only offered to senior endosonographers who are deemed suitable and sufficiently experienced. By renewing the pool of practitioners who are willing to take on EUS training and mentoring responsibilities, EUS education can be propagated further using the existing short‐term training programs that AEG provide. To date, there have been more than 40 trainers who have successfully emerged through the programs.^29^

## Conclusion

Given the expanding indications and therapeutic capabilities of EUS, the demand for competent endosonographers will only keep increasing. Education and training of EUS in most parts of Asia are thus likely to remain a formidable challenge for years to come. Ultimately, there needs to be a change in mindset on how endosonographers should be trained, and a diverse range of training programs need to be accommodated to help Asia cope with the scarcity of formal EUS training fellowships. The progressive learning model created by AEG appears to hold the key to helping fill the vacuum in regional EUS training. The value of its training programs, however, can reach its full potential only if these programs are officially endorsed and its program graduates can transition to formal clinical apprenticeship to achieve the credentials required for independent EUS practice.
